# Accelerated Resolution Therapy for treatment of pain secondary to symptoms of combat-related posttraumatic stress disorder

**DOI:** 10.3402/ejpt.v5.24066

**Published:** 2014-05-07

**Authors:** Kevin E. Kip, Laney Rosenzweig, Diego F. Hernandez, Amy Shuman, David M. Diamond, Sue Ann Girling, Kelly L. Sullivan, Trudy Wittenberg, Ann M. Witt, Cecile A. Lengacher, Brian Anderson, Susan C. McMillan

**Affiliations:** 1 College of Nursing, University of South Florida, Tampa, FL, USA; 2 Western New England University, Springfield, MA, USA; 3 Department of Psychology, University of South Florida, Tampa, FL, USA; 4 Department of Molecular Pharmacology and Physiology, Center for Preclinical/Clinical Research on PTSD, University of South Florida, Tampa, FL, USA; 5 Morsani College of Medicine, University of South Florida, Tampa, FL, USA; 6 PieWiseLiving, LLC, Tampa, FL, USA; 7 Pasco County Veterans Service Office, Port Richey, FL, USA

**Keywords:** Psychological trauma, PTSD, pain, exposure therapy, psychotherapy, imagery rescripting, prevalence, clinical trials, eye movements, combat

## Abstract

**Background:**

As many as 70% of veterans with chronic pain treated within the US Veterans Administration (VA) system may have posttraumatic stress disorder (PTSD), and conversely, up to 80% of those with PTSD may have pain. We describe pain experienced by US service members and veterans with symptoms of PTSD, and report on the effect of Accelerated Resolution Therapy (ART), a new, brief exposure-based therapy, on acute pain reduction secondary to treatment of symptoms of PTSD.

**Methods:**

A randomized controlled trial of ART versus an attention control (AC) regimen was conducted among 45 US service members/veterans with symptoms of combat-related PTSD. Participants received a mean of 3.7 sessions of ART.

**Results:**

Mean age was 41.0 + 12.4 years and 20% were female. Most veterans (93%) reported pain. The majority (78%) used descriptive terms indicative of neuropathic pain, with 29% reporting symptoms of a concussion or feeling dazed. Mean pre-/post-change on the Pain Outcomes Questionnaire (POQ) was −16.9±16.6 in the ART group versus −0.7±14.2 in the AC group (*p*=0.0006). Among POQ subscales, treatment effects with ART were reported for pain intensity (effect size = 1.81, *p*=0.006), pain-related impairment in mobility (effect size = 0.69, *p*=0.01), and negative affect (effect size = 1.01, *p*=0.001).

**Conclusions:**

Veterans with symptoms of combat-related PTSD have a high prevalence of significant pain, including neuropathic pain. Brief treatment of symptoms of combat-related PTSD among veterans by use of ART appears to acutely reduce concomitant pain.

By 2013, more than 51,000 individuals in the US military were wounded in action in the recent Operation Iraqi Freedom (OIF), Operation Enduring Freedom (OEF), and Operation New Dawn (OND) conflicts combined (Iraq and Afghanistan Veterans of America, 2013), and the less visible psychological wounds of war continue to be a problem (Tanielian & Jaycox, 2008). It is estimated that as many as 70% of veterans with chronic pain treated within the US Veterans Administration (VA) system may have posttraumatic stress disorder (PTSD), and conversely, up to 80% of those with PTSD may have pain (Beckham et al., 1997; Lew et al., 2009; Otis, Keane, & Kerns, 2003; Shipherd et al., 2007; Stecker, Fortney, Owen, McGovern, & Williams, 2010). Patients with both PTSD and chronic pain generally present with more complicated clinical profiles (Sharp & Hanery, 2001), and no formal treatment guidelines exist for comorbid PTSD and chronic pain (Muller et al., 2009). Such individuals report much lower quality of life, and the presence of chronic pain may serve as a constant reminder of a traumatic event and may also worsen PTSD symptoms (U.S. Department of Veterans Affairs, 2007). Further, veterans with PTSD receive more frequent and higher-dose opioids for pain diagnoses (Seal et al., 2012). Use of prescription opioids for pain is associated with risk of alcohol-, drug-, and opioid-related accidents/overdoses, as well as self-inflicted injuries (Seal, et al., 2012).

Pain may be categorized by type, including somatic, visceral, and neuropathic (Fig. 1), and as used in the present analysis. Somatic pain is associated with the musculoskeletal tissues, is localized, and is often described as constant, aching, or pulling. Visceral pain is experienced in the internal organs, is vague, poorly defined, not localized, and may be experienced as squeezing or cramping pain. Neuropathic pain, irrespective of disease condition, is related to nerve involvement and is often described as burning, stabbing, or stinging pain, or as pins and needles (Levy, Chwistek, & Mehta, 2008). Pain is most appropriately treated by type and severity.

**Fig. 1:**
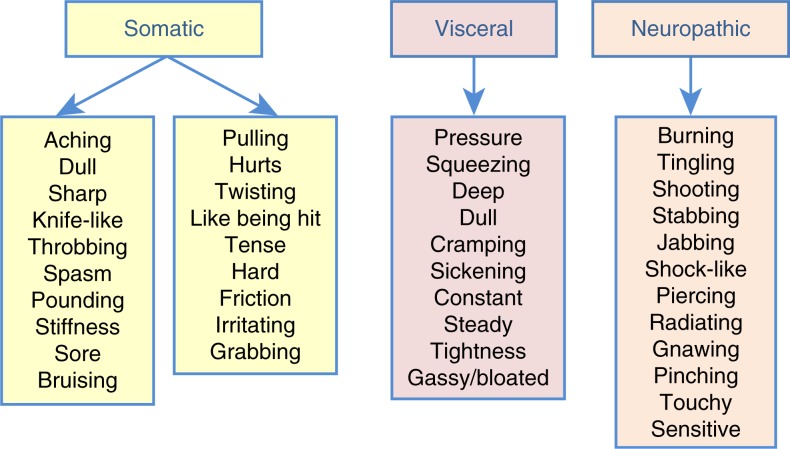
Descriptive terms used to classify origin of reported pain as somatic, visceral, and/or neuropathic among all study participants.

Musculoskeletal and connective system ailments involving back, neck, head, and abdominal pain are some of the most frequent reasons that veterans seek care at the VA (Gironda, Clark, Massengale, & Walker, 2006; Haskell et al., 2012; Kang, Mahan, Lee, Magee, & Murphy, 2000; Lew, et al., 2009; Taylor et al., 2012). Evidence-based therapies serve as separate first-line treatments for PTSD and pain in veterans, but these therapies are only partially effective. Therapies used frequently for PTSD include prolonged exposure (PE) therapy and cognitive processing therapy (CPT) (U.S. Department of Veterans Affairs, 2008), both of which are lengthy, costly, and result in highly variable rates of dropout and treatment success; cognitive behavioral therapies (CBT) for treatment of pain show mixed results (Basler, Jakle, & Kroner-Herwig, 1997) and only medium effect sizes (Morley, Eccleston, & Williams, 1999). Moreover, use of opioid analgesics by veterans with PTSD requires special attention because of the potential for addiction and fatal overdose either by accident or attempted suicide (U.S. Department of Veterans Affairs, 2009). In addition to these existing therapies, there is a new approach called Accelerated Resolution Therapy (ART), an evidence-based psychotherapy that is delivered in 2–5 sessions and without the need for or use of medications. As part of a randomized controlled trial (RCT) of the effect of ART on symptoms of combat-related PTSD (Kip, Rosenzweig, et al., 2013), the investigators collected data on pain, and, thus, were able to conduct a secondary analysis that shed light on the effectiveness of ART for pain management in service members/veterans with symptoms of PTSD. While results have indicated statistically and clinically significant reductions in symptoms of PTSD in US civilians (Kip et al., 2012; Kip, Sullivan, et al., 2013) and, more recently, in service members and veterans (Kip, Rosenzweig, et al., 2013), it was unknown whether ART would improve pain symptoms. Thus, the purpose of this paper is to describe the pain experienced by veterans with symptoms of PTSD and to report the acute effect of ART on that pain.

## Methods

### Study design

A two-group RCT was conducted in which consenting and eligible service members and veterans (described below) were randomly assigned to treatment with ART or an attention control (AC) regimen. Study participants randomly assigned to AC were offered treatment (crossover) with ART upon completion of the AC regimen. The Institutional Review Board (IRB) at the University of South Florida and the DoD Telemedicine and Advanced Technology Research Center (sponsor of the trial) approved the trial protocol. All participants provided written informed consent, and the trial was registered with ClinicalTrials.gov (NCT01559688).

### Recruitment

Participants were recruited from community-based organizations and veteran membership organizations within the Tampa Bay area, as well as through academic programs at the University of South Florida (USF). Referrals for study participation were provided by the James A. Haley VA Hospital (Tampa, FL), Bay Pines VA Hospital (Bay Pines, FL), and United States Special Operations Command (USSOCOM), Care Coalition, MacDill Air Force Base (Tampa, FL). Participants recruited from these sources who received ART and/or the AC regimens were evaluated and treated at the USF College of Nursing, Tampa, FL.

### Screening

Clinical evaluation used for the parent trial eligibility consisted of the 17-item PTSD Checklist, Military Version (PCL-M Checklist), 125-item Psychiatric Diagnostic Screening Questionnaire (PDSQ), Brief Mental Status Exam, and self-developed nine-item ART Intake Questionnaire. The PCL-M Checklist is a self-report of DSM-IV symptoms of PTSD in response to stressful military experiences (Blanchard, Jones-Alexander, Buckley, & Forneris, 1996; Weathers, Litz, Herman, Huska, & Keane, 1993) and is used with service members and veterans. The PDSQ was used to screen for Axis I disorders to serve as a baseline assessment of psychopathology (Zimmerman & Chelminski, 2006; Zimmerman & Mattia, 2001). The nine-item ART Intake Questionnaire is designed to capture information on traumas impacting the veterans including the number of traumatic events, duration of symptoms, self-reported guilt, and prior treatment. Completion and scoring of the PCL-M and PDSQ was followed by clinical ART clinician/veteran interviews to determine study eligibility.

Trial inclusion criteria were: (1) US service member or veteran with prior deployment(s); (2) age≥18 years; (3) symptoms of psychological trauma including score ≥40 on the PCL-M Checklist and/or endorsement of PTSD items on the PDSQ; (4) ability to read and speak English (eighth-grade level) in order to complete survey questions; (5) denial of suicidal or homicidal ideation; and (6) no evidence of psychotic behavior or psychological crisis. Exclusion criteria consisted of: (1) brain injury prohibiting speech, writing, and purposeful actions; (2) major psychiatric disorder (e.g., bipolar disorder) concomitant to symptoms of psychological trauma (as defined above); (3) currently undergoing substance abuse treatment; (4) previous diagnosis of eye movement disorder sufficient to interfere with treatment, as anticipated by the ART clinician; and (5) any medical condition that, in the judgment of the principal investigator and/or ART clinician, might place the individual at risk due to a potential reaction (e.g., previous heart attack, seizure disorder).

### Random assignment

Eligible service members/veterans were randomly assigned to the ART or AC regimen in a 1:1 ratio using a random number generator and variable blocking scheme (blocks of 4, 6, and 8). The first session (ART or AC) was typically scheduled within 1 week (usually sooner) of screening.

### ART intervention

The ART intervention, delivered in 2–5 sessions each approximately 60–75 min in duration, consisted of two components and the use of bilateral eye movements. In the first component, *Imaginal Exposure* (IE) was used whereby participants were asked to recall (verbally or non-verbally) the traumatic event (scene) while focusing on physiological sensations, thoughts, and emotions. During this process, the participant, with coaching from the ART clinician, was composed into a relaxed, alert state of mind and then exposed to re-activation of the targeted memory for a short 30–45 s period of time. This period of exposure to the memory was followed by identification and diminishment (or eradication) of any uncomfortable emotional or somatic symptoms.

In the second component, *Imagery Rescripting* (IR) was used whereby participants were instructed to visualize their traumatic scene and imagine changing (replacing) the imagery and sensory components of the scene to any positive scene of their choice. As the new positive scene was then substituted and reviewed, the participant was asked to try to access the original distressing images. Treatment of the traumatic scene was considered complete (successful) when only the replacement scene could be accessed, although knowledge of the original scene remained in memory. The number of ART sessions per patient was variable and was based on processing (treating) the specific, number of traumatic scenes identified by the patient as contributing to symptoms of PTSD.

Throughout components and sensation checks of the therapy, the participant was asked to follow the therapist's hand back and forth moving the eyes from left to right, with 40 eye movements per set. During this process, the participants were not speaking but, rather, “watching” their original or newly imagined scene. This process of “watching” the scene (during both IE and IR) while performing eye movements was repeated multiple times, with the total sets of eye movements determined by the number required to complete the IE and IR components. Additional details on the ART protocol have been published (Kip, et al., 2012, 2014; Kip, Rosenzweig, et al., 2013; Kip, Sullivan, et al., 2013).

### AC intervention

The AC intervention consisted of two one-hour sessions of fitness assessment and planning or two one-hour sessions of career assessment and planning, as selected by the service member/veteran. The fitness assessment and planning regimen was conducted by a certified health fitness trainer. The assessment included anthropometric measures, determination of body fat percentage and body mass index, review of previous exercise history, and identification of individualized physical fitness goals. The career assessment and planning regimen was conducted by a professional career counselor. It included completion and review of the Career Planning Scale which encompasses six scales covering knowledge of the world of work, knowledge of occupations, self-knowledge, career decision-making, career planning, and career implementation (Liptak, 2001).

### Data collection

After screening and enrollment in the trial, participants completed a demographic and brief medical history questionnaire. In addition, baseline completion of self-reported outcome measures (in addition to the previously completed PCL-M) included the following measures: The 20-item Center for Epidemiologic Studies Depression Scale (CES-D) (Radloff, 1977); 18-item Brief Symptom Inventory (BSI) (Derogatis, 2001); 21-item State-Trait Inventory for Cognitive and Somatic Anxiety (STICSA) (Ree, French, MacLeod, & Locke, 2008); Pittsburgh Sleep Quality Index (PSQI) (Buysse, Reynolds, Monk, Berman, & Kupfer, 1989); 32-item Trauma-Related Guilt Inventory (TRGI) (Kubany, 1996); 21-item Post-Traumatic Growth Inventory (PTGI) (Tedeschi & Calhoun, 1996); 26-item Self-Compassion Scale (SCS) (Neff, 2003); 29-item Aggression Questionnaire (AQ) (Buss & Perry, 1992); and the10-item Alcohol Use Disorder Identification Test (AUDIT) (Saunders, Aasland, Babor, de la Fuente, & Grant, 1993). As part of PTSD comorbidity evaluation, participants also completed the 20-item Pain Outcomes Questionnaire (POQ)—Short Form (Clark, Gironda, & Young, 2003). This reliable and valid instrument contains 19 primary pain items that are rated on an 11-point (0–10) Likert-type scale and one demographic question. In addition to a total pain score, six subscale scores can be calculated that correspond to: pain intensity (one item), pain-related impairment in mobility (four items), pain-related impairment in performing activities of daily living (four items), sense of impairment in activity and energy levels (three items), dysphoric affect and associated symptoms (five items), and pain-related fear and avoidance (two items). Participants received $50 each time they completed the set of study assessments (pre-ART, post-ART, and at 3-month follow-up). Despite having a 3-month posttreatment follow-up, this report pertains to the acute effect of ART on pain (i.e., pre-ART vs. post-ART), since after crossover to ART by the control group, no randomized comparison was possible at the 3-month follow-up.

### Statistical methods

**Fig. 2:**
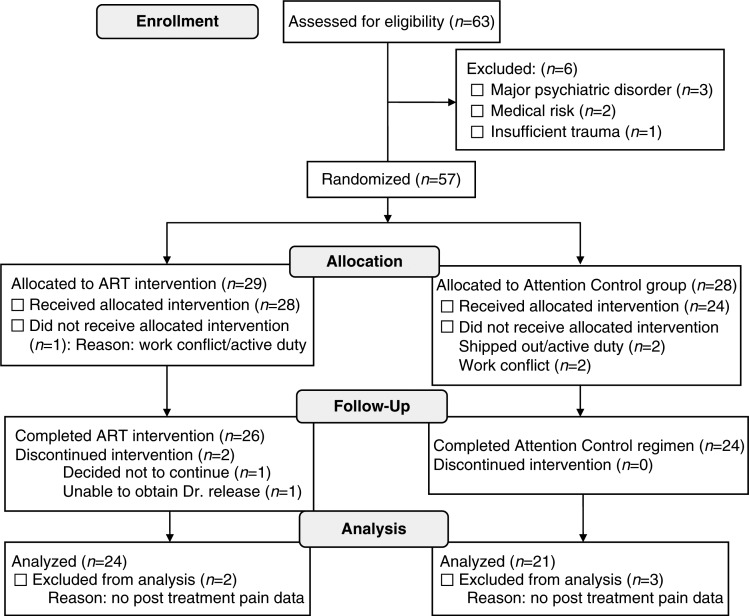
Consort diagram of the trial population including those screened, enrolled, randomly assigned, completing treatment, and analyzed.

Demographic, military, and clinical characteristics of the study sample are described by means and standard deviations for continuous variables and percentages for categorical variables. Of the 57 participants randomly assigned (see Fig. 2), 45 provided pain outcome data before and after their assigned regimen. Thus, distributions of baseline characteristics were first compared between those with and without pain outcome data, followed by comparisons by random assignment by use of Student *t*-tests and Fisher's exact test. For the study outcome of change in pain scores on the POQ, analysis of covariance (ANCOVA) was used to compare mean pre-/post-differences by random assignment, adjusting for the baseline value. Standardized effect sizes for pain scores were calculated as: ([mean before ART - mean after ART]/standard deviation of treatment difference scores) (Morris & DeShon, 2002). Pearson correlation coefficients were calculated to assess the strength of relationship between symptoms of PTSD and pain. Given the exploratory nature of the analysis, a two-sided *p*-value of <0.05 was used to define statistical significance in all analyses, without adjustment for multiple comparisons. For total score on the POQ, an intention to treat (ITT) analysis was conducted assuming no difference (value of 0) in pain scores among the 12 of 57 participants without complete pain data. Finally, while not defined *a priori*, results were examined by two subgroups of interest. This included: (1) whether the primary trauma for which treatment was sought was classified as physical (consisting of military sexual trauma, improvised explosive device blast or combat explosion, or three or more traumas) versus psychological (consisting of witnessing of death, execution or major injuries, or homicide of civilians) and (2) among those with versus without a history of head trauma.

## Results

### Sample

A total of 63 service members/veterans were assessed for trial eligibility, of whom, 57 (90.5%) were eligible and enrolled (Fig. 2). Of the 57 participants enrolled, 29 (50.9%) were assigned to the ART intervention and 28 (49.1%) were assigned to the AC intervention. A total of 52 of the 57 participants (91.2%) received their assigned regimen, of whom, 50 (96.2%) completed their assigned regimen. Of these 50 participants, 45 (90%) provided pre- and post-intervention pain score data, providing the basis for the analysis. The five participants with missing post-intervention data did not complete the POQ. Presenting characteristics were similar between the 45 participants with and 12 participants without pre- and post-intervention pain outcome data. Apparent exceptions were the 45 participants with pain data being more likely discharged veterans as opposed to active duty or reservist (75.6% vs. 50.0%, *p*=0.18), and having received prior treatment for PTSD (73.3% vs. 50.0%, *p*=0.17).

Among the 45 participants in the study, the mean age was 41.0 ± 12.4 years, 20% were female, 84.4% were of Caucasian race, 55.6% had primary military service in the Army, 44.4% were on disability for PTSD or another mental health disorder, and 46.7% had lived with traumatic memories for more than 10 years. The mean PTSD symptom score on the PCL-M was 56.9±14.9 and mean total pain score on the POQ was 50.5±29.4. Demographic, military, and clinical characteristics were generally similar by random assignment (Table 1). Exceptions were the ART group (compared to AC group) having a higher prevalence of veterans (non-active duty) (75.6% vs. 50.0%, *p*=0.03) and Hispanic representation (20.8% vs. 0.0%, *p*=0.05). Of note, presenting PTSD symptom score on the PCL-M (*p*=0.90) and total pain score on the POQ (*p*=0.81) were similar by random assignment.

**Table 1:** Demographic, military, and clinical characteristics by random assignment

Characteristic	AC (*n*=21)	ART (*n*=24)	*p*
Age in years (mean±SD)	44.0±13.5	38.4±10.9	0.14
Female gender (%)	19.1	20.8	1.0
Race (%)			0.95
White	85.7	83.3	
Black or African American	9.5	12.5	
Other	4.8	4.2	
Hispanic ethnicity (%)	0.0	20.8	0.05
Current military status (%)			0.18
Active duty	16.7	11.1	
Reservist	33.3	13.3	
Discharged/veteran	50.0	75.6	
Primary branch of military service (%)			0.03
Army	42.9	66.7	
Navy	14.3	25.0	
Air Force	19.0	8.3	
Marines	23.8	0.0	
On disability for PTSD/other MH disorder	33.3	54.2	0.23
Five or more traumatic memories currently impacting life (%)	38.1	50.0	0.55
Lived with traumatic memories >10 years (%)	52.4	41.7	0.56
Previous treatment for PTSD (%)	76.2	70.8	0.75
Individual therapy	71.4	58.3	0.53
Group therapy	28.6	20.8	0.73
Pharmacotherapy	52.4	62.5	0.56
PCL-M score (mean±SD)	56.6±15.0	57.2±15.1	0.90
PCL-M score ≥50 (%)^a^	57.1	70.8	0.37
PCL-M critical items for PTSD (%)^b^	71.4	79.2	0.73
PDSQ score (mean±SD) (T-score)	54.4±11.7	54.0±9.5	0.90
Any PTSD screening criteria (%)^c^	85.7	95.8	0.33
POQ scores (mean±SD)			
Pain intensity	3.8±2.9	3.8±2.3	0.98
Pain-related impairment in mobility	9.2±11.4	8.8±10.2	0.89
Pain-related impairment in completing ADLs	2.8±5.9	3.1±7.4	0.87
Vitality—impairment in activity/energy	14.8±6.3	15.4±12.6	0.76
Negative affect	21.5±10.5	20.3±10.6	0.70
Pain-related fear and avoidance	−0.4±4.0	−1.9±2.8	0.16
Total POQ score	51.6±38.2	49.5±29.9	0.81

PDSQ: Psychiatric Diagnostic Screening Questionnaire; PCL-M: PTSD Checklist, Military Version; POQ: Pain Outcomes Questionnaire.

a Established screening cutpoint score for probable PTSD.

b DSM-IV symptom criteria for probable PTSD, at least one “B” item (questions 1–5), three “C” items (questions 6–12), and at least two “D” items (questions 13–17) rated as “Moderate” or above.

c Screening criteria for PTSD from the PCL-M, and/or PDSQ.

### Presenting injuries and pain

As part of the clinician intake and documentation process, participants were asked about the kinds of injuries or problems they were having (i.e., not a self-report questionnaire). The largest number (29%) reported having symptoms of a concussion or feeling dazed, or, similarly, having experienced a traumatic brain injury (TBI) or head injury (22%) (Table 2). Many reported multiple problems, and 16% reported tinnitus. Most participants in the sample (93%) reported pain of some type, and approximately half (46.7%) reported pain intensity at a level of four or higher, with some respondents reporting pain scores as high as 9 on a 0 − 10 scale. Including all participants in the sample, the mean pain score was 3.8 (SD = 2.6); this mean included the four participants who reported no pain. When asked to describe the pain they were experiencing at the clinician intake, the majority (77.8%) used descriptive terms that would normally characterize neuropathic pain. This was much higher than terms used to characterize pain as somatic (26.7%), visceral (8.9%), or of multiple types (26.7%).

**Table 2:** Frequency and percent of types of injuries and problems reported by service members and veterans (*N*=45)

Type of injury or problem	Frequency	Percent*
Concussion or dazed	13	29
TBI or head injury	10	22
Arm or leg injury or pain	7	16
Ringing in the ears	7	16
Headaches or migraines	4	9
Dizziness or vertigo	3	7
Memory problems	3	7
Other problems: Paraplegia, fibromyalgia, Meniere's disease, irritability	4	9

* Participants reported more than one problem, so totals add up to more than 100%.

### Effect of ART on change in pain scores

**Fig. 3:**
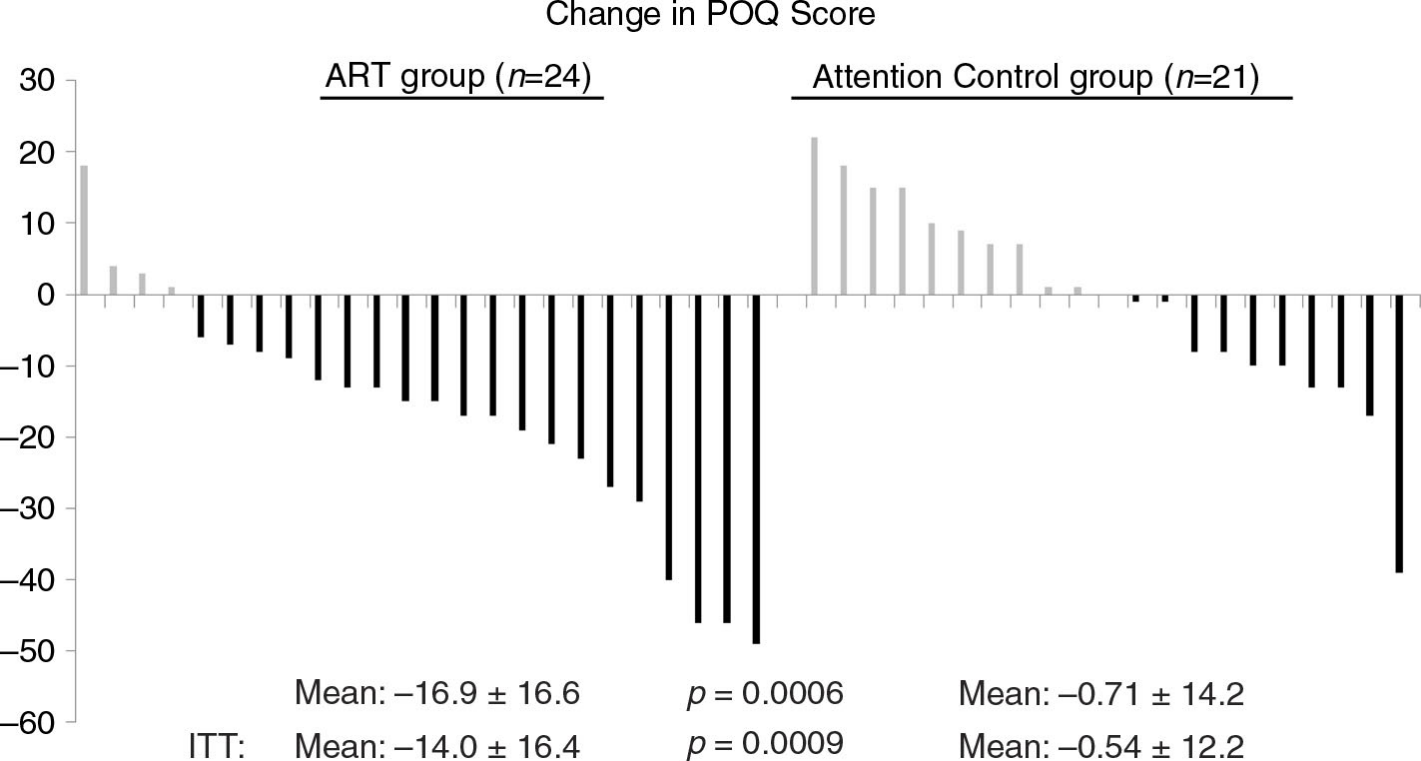
Plot of change scores on the Pain Outcomes Questionnaire (POQ) before and after treatment with Accelerated Resolution Therapy (ART) versus before and after an attention control (AC) regimen. Each vertical line represents the response of an individual service member or veteran. ITT=intention to treat analysis.

The 24 participants assigned to ART who completed treatment for symptoms of PTSD underwent a mean of 3.7±1.0 sessions. All 21 participants assigned to the AC group who initiated the intervention completed two sessions (per study protocol). Among the 45 completers of their randomly assigned intervention, the mean pre-/post-change on the POQ was −16.9±16.6 in the ART group versus −0.7±14.2 in the AC group (effect size=1.04, *p*=0.0006) (Fig. 3, Table 3). In the ITT analysis (*n*=57), the mean pre-/post-change on the POQ was −14.0±16.4 in the ART group versus −0.5±12.2 in the AC group (*p*=0.0009). Among the POQ subscales, significant, acute treatment effects associated with ART were reported for pain intensity (effect size=1.81, *p*=0.006), pain-related impairment in mobility (effect size=0.69, *p*=0.01), negative affect (effect size=1.01, *p*=0.001), and in a counter-direction, pain-related fear and avoidance (effect size=−0.87, *p*=0.02). Due to potential floor effects (i.e., limited pain at baseline), analyses were repeated among the 21 participants with a pain intensity score of 4 or more prior to intervention (Table 4). In this subset, the mean pre-/post-change on the POQ was −21.3±20.4 in the ART group versus 1.6±12.0 in the AC group (effect size = 1.32, *p*=0.004). In the ART group, there was no discernable pattern of mean reduction of total POQ scores by the number of ART sessions received: two (*n*=3,−22.0±24.5); three (*n*=7, −7.1±13.6); four (*n*=8, −16.2±15.8); and five (*n*=6, −26.7±14.0).

**Table 3:** Mean pre- to post-assessment differences in POQ scale score by random assignment (all participants)

	AC (*n*=21)		ART (*n*=24)		Between group			
	
POQ scale	Mean*	SD	Mean*	SD	Mean*	SD	Effect size	p**
Pain intensity	−0.29	1.59	1.17	1.99	1.45	1.81	0.80	0.006
Mobility	−0.45	6.66	3.88	5.94	4.33	6.29	0.69	0.01
ADL	−0.05	3.32	1.75	4.72	1.80	4.11	0.44	0.10
Vitality	−0.91	4.82	2.79	8.10	3.70	6.74	0.55	0.06
Negative affect	2.18	6.25	9.50	8.10	7.32	7.27	1.01	0.001
Fear	0.55	2.34	−2.17	3.67	−2.71	3.11	−0.87	0.02
Total POQ score	0.71	14.15	16.92	16.62	16.20	15.52	1.04	0.0006

AC: attention control regimen; ART: Accelerated Resolution Therapy intervention; POQ: Pain Outcomes Questionnaire.

* Positive values indicate reductions in POQ scale scores.

** Adjusted for baseline value.

**Table 4:** Mean pre- to post-assessment differences in POQ scale score by random assignment (participants with a pain score of four or more at study entry)

	AC (*n*=9)		ART (*n*=12)		Between group			
	
POQ scale	Mean*	SD	Mean*	SD	Mean*	SD	Effect size	p**
Pain intensity	0.56	1.13	2.25	1.76	1.69	1.53	1.11	0.08
Mobility	−3.00	8.02	7.08	6.71	10.08	7.29	1.38	0.002
ADL	−0.56	5.22	3.58	6.22	4.14	5.82	0.71	0.05
Vitality	−0.67	4.24	2.58	10.43	3.25	8.40	0.39	0.35
Negative affect	1.56	5.90	7.75	6.97	6.19	6.54	0.95	0.07
Fear	0.56	1.67	−1.92	4.27	−2.47	3.43	−0.72	0.07
Total POQ score	−1.56	12.03	21.33	20.36	22.89	17.35	1.32	0.004

AC: attention control regimen; ART: Accelerated Resolution Therapy intervention; POQ: Pain Outcomes Questionnaire.

* Positive values indicate reductions in POQ scale scores.

** Adjusted for baseline value.

### Subgroup analyses

Among participants with their primary trauma classified as of physical origin (*n*=26), the mean pre-/post-change on the POQ was −19.6±17.7 in the ART group versus −1.3±14.9 in the AC group (effect size=1.11, *p*=0.01). This compared to −13.2±15.0 in the ART group versus 0.1±13.9 in the AC group among participants with primary psychological trauma (*n*=19, effect size = 0.92, *p*=0.02). The test for effect modification (interaction) was not significant (*p*=0.81). Among participants without a history of head trauma (*n*=31), the mean pre-/post-change on the POQ was −15.2±19.7 in the ART group versus −0.7±15.6 in the AC group (effect size=0.82, *p*=0.01). This compared to −19.8±9.9 in the ART group versus −0.8±9.7 in the AC group among participants with a history of head trauma (*n*=14, effect size = 1.92, *p*=0.005). Although the effect size associated with ART appeared markedly higher among participants with a history of head trauma, the formal test for effect modification was not significant (*p*=0.58).

### Relationship between PTSD and pain

**Fig. 4:**
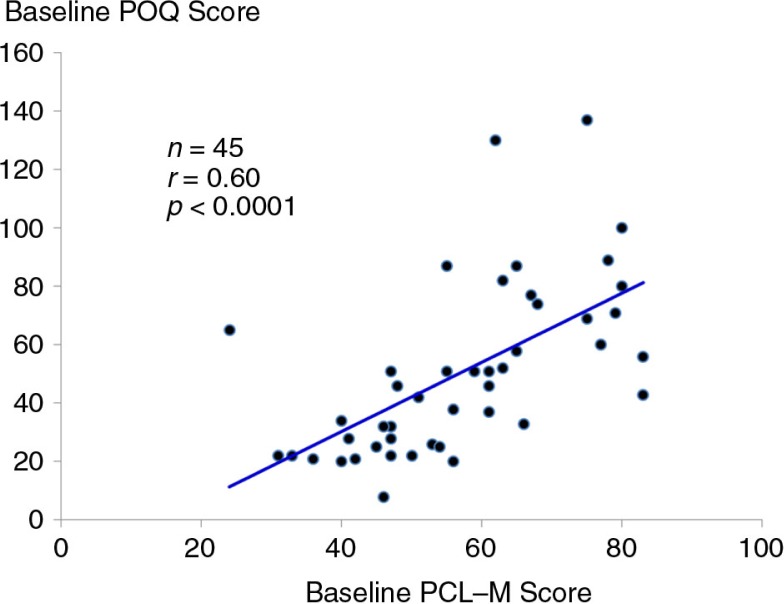
Scatter plot and linear regression line of the relationship between baseline PTSD symptom score from the PCL-M and baseline total pain score from the POQ.

**Fig. 5:**
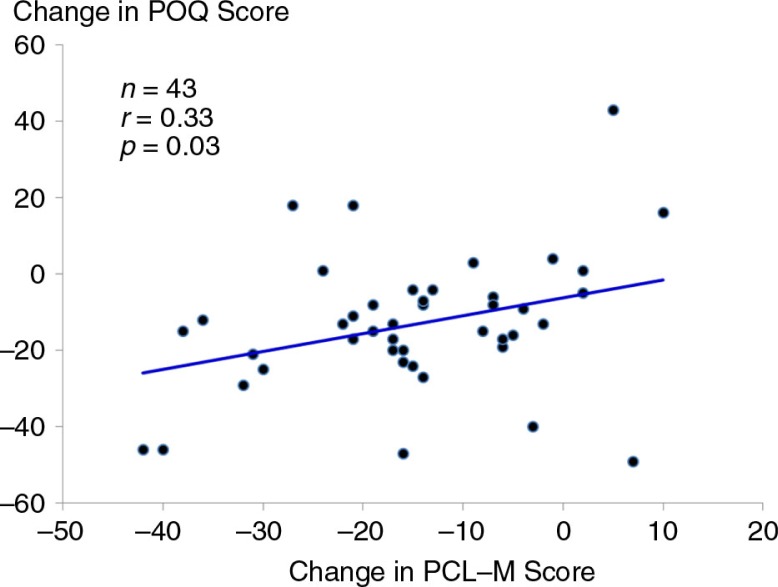
Scatter plot and linear regression line of the relationship between change in PTSD symptom score from the PCL-M and change in total pain score from the POQ before and after treatment with Accelerated Resolution Therapy (ART).

At baseline, there was a strong, positive correlation between symptoms of PTSD measured from the PCL-M and total pain scores on the POQ (*r*=0.60, *p*<0.0001) (Fig. 4). For the 43 participants who completed treatment with ART (i.e., irrespective of random assignment), pre- to post-changes in symptoms of PTSD were positively associated with changes in pain scores on the POQ (*r*=0.33, *p*=0.03) (Fig. 5).

## Discussion

In this RCT designed to evaluate treatment of symptoms of PTSD, two interesting, and, to some extent, unexpected findings were observed. First, the extent and severity of comorbid pain among US service members/veterans presenting for treatment of symptoms of PTSD was substantial. Second, treatment of symptoms of PTSD with the use of ART appeared to generalize substantially to meaningful, acute reductions in pain.

### Prevalence of pain

Whereas study participants described their injuries or problems using their own words, there was much similarity in their descriptions. Because of the percent (22%) reporting TBI or head injury, it is not surprising that having symptoms of concussion or feeling dazed was the most common (29%) response. What was not expected was that the vast majority (93%) of the participants referred for treatment of symptoms of PTSD also had pain. Moreover, almost half (47%) reported pain at a level of 4 or higher on a 0 − 10 scale. Thus, while symptoms of PTSD were a significant problem for them and one for which they were seeking treatment, it was not their only problem in need of treatment. Many of these service members/veterans had significant pain that was intense enough to impair quality of life and possibly complicate the treatment of symptoms of PTSD.

Unfortunately, health care providers often do not administer opioid medications in sufficient doses to relieve pain completely (Broekmans, Vanderschueren, Morlion, Kumar, & Evers, 2004), and opioids are contraindicated in the presence of some comorbidities, including acute psychiatric instability and diagnosed substance use disorder (non-nicotine) not in remission and not in treatment (US Department of Veterans Affairs, 2010). In fact, earlier research with veterans found that among 90 veteran inpatients, pain was poorly assessed and poorly managed (McMillan, Tittle, Hagan, & Laughlin, 2000). Although these service members and veterans in our sample were experiencing real pain as a result of real injuries, there is a strong likelihood that their pain was not being adequately managed because of lack of knowledge about analgesics and fear of opioid-related side effects on the part of health care providers (Edwards et al., 2001). Such fear and knowledge deficits have a negative effect on how analgesics are administered, leading to mis-managed pain treatment and patient suffering (Broekmans, et al., 2004). In addition, many health care providers, because of their poor understanding of opioids and patients in pain, may label a patient as “drug-seeking” if he or she seeks analgesics for pain relief (McCaffery, Grimm, Pasero, Ferrell, & Uman, 2005).

### Types of pain

The majority of study participants (78%) described their pain using terms suggestive of neuropathic pain. This likely relates to the different kinds of physical damage done by wounds of war, and points out the need for careful assessment of the types of pain that service members and veterans experience in order to develop better treatment protocols. In general, neuropathic pain is better managed with anti-convulsant or anti-depressant medications rather than opioids, adding opioids principally when the veteran is having mixed types of pain. Wounds that occur in battle might logically be bodily injury to bones and soft tissue, leading to somatic pain. However, only 16% of participants reported injuries to arms and legs, and only 27% used terms that would normally be used to describe somatic pain. This finding may be the result of the bias in the way the group was accrued to the study; that is, all of these US service members/veterans had symptoms of PTSD and were not specifically referred because of the type of injury that they had experienced. The small number of somatic injuries compared to the much larger number of concussions, feeling dazed, TBI and head injuries probably accounted for the high prevalence of neuropathic pain descriptors. The lowest percentage of participants used the terms that described visceral pain. This is probably expected in a relatively young population. Visceral pain is typically seen in angina, kidney colic, or colitis, conditions that are not typically prevalent in young service members and veterans.

### Changes in pain scores

Although pain was not the focus of the original clinical trial, pain data collected before and after the ART intervention allowed for this analysis. Thus, the finding that the service members/veterans in the ART arm of the trial had significantly greater reduction in pain scores is quite striking. Pain intensity, which is the score that most patients and health care providers focus on when assessing for pain, showed a significantly (*p*=0.006) greater reduction in the ART group compared to the AC group. When all subscales of the POQ were summed and compared, the mean difference between groups was substantial (−16.2±15.5 points), and highly statistically significant (*p*=0.0006), including in the ITT analysis (*p*=0.0009). This finding using the total POQ scores probably occurred because the subscales on the POQ other than pain intensity all could be affected by mood states such as PTSD. Thus, when the symptoms of PTSD improved as a result of ART, the subscales assessing negative affect and vitality might be expected to improve as well.

### Possible mechanism

An unknown yet signature question from this analysis centers on the possible mechanism by which ART, an exposure-based psychotherapy used to treat symptoms of PTSD, appears to result in favorable concomitant reductions in pain. Importantly, during the IE phase of the ART sessions, participants were directed to focus exclusively on physiological sensations elicited from recall of the traumatic experience. In many instances, recall of the psychological trauma was directly linked to adverse pain experiences. Such physiological sensations were then “processed out” (diminished or eliminated) through repeated sets of eye movements. Still, how is it that removing physiological sensations elicited from recall of previous traumas may conceivably generalize to reductions in chronic pain at large?

There is evidence that psychological trauma induces change in biological substrates, which alter both pain transduction pathways and pain processing mechanisms in the brain (Geuze et al., 2007; Liberzon et al., 2007). However, the manner in which treatment of PTSD influences such bidirectional relationships is unclear. Specifically, clinical studies report that pain experience in persons with PTSD is significantly increased compared with control subjects (Asmundson, Coons, Taylor, & Katz, 2002; Beckham, et al., 1997; Defrin et al., 2008). Paradoxically, empirical research also indicates that patients with PTSD report a decrease in pain intensity ratings after exposure to traumatic reminders and temperature-induced pain assessment (Geuze, et al., 2007; Kraus et al., 2009; Pitman, van der Kolk, Orr, & Greenberg, 1990). Still, there is limited evidence that trauma-focused exposure therapy reduces anxiety and physiological arousal, and, in turn, decreases pain severity and general distress (Dunne, Kenardy, & Sterling, 2012; Jaspers, 1998; Wald, Taylor, Chiri, & Sica, 2010).

PTSD is characterized by hyperactivation of the amygdala and hippocampus, and lower activation and imbalance in the medial prefrontal cortex (Patel, Spreng, Shin, & Girard, 2012; Vermetten & Bremner, 2002). Of note, the amygdala integrates nociceptive information and plays a dual facilitatory and inhibitory role in the modulation of emotional pain behavior (Neugebauer, Li, Bird, & Han, 2004). An entirely theoretical hypothesis is that changing of images and sensations in the imagery rescripting component of the ART protocol “corrects” disinhibition of the amygdala that is present in PTSD and, similarly, through the process of reconsolidation (Monfils, Cowansage, Klann, & LeDoux, 2009), breaks the direct brain-based association between the trauma and concomitant pain.

However, our data showed no evidence of ART being more effective in acutely reducing pain when the principal trauma being treated included physical injury, as opposed to being primarily psychological in origin. Thus, an alternative, more systemic hypothesis is that improvement of PTSD symptoms with ART, especially reduction in sleep disturbance which is exceptionally prevalent in PTSD patients (Maher, Rego, & Asnis, 2006), may result in the secondary benefits of normalized immune function and reduced somatization, and, therefore, reduced pain (Gupta, 2013). Clearly, future neuroimaging studies are required to elucidate how exposure-based therapies, including ART, may generalize to concomitant reduction in pain.

### Strengths and limitations

Strengths of the study include the use of a highly standardized treatment protocol (ART), a wide range of therapists with different backgrounds to enhance the generalizability of treatment delivery, and the exclusion of involvement by the founder or lead ART trainer with any outcome assessment, to eliminate potential ascertainment bias. A principal limitation is that the ART intervention was not designed (or delivered) specifically for pain reduction concomitant to symptoms of PTSD. Thus, theoretical explanations for our results range from a possible spurious association (i.e., no true effect of ART on pain reduction) to a potential underestimation of the effect of ART on pain reduction (i.e., had the intervention been tailored and delivered specifically for pain). In addition, the ART intervention was not compared to an active psychotherapy or otherwise pain reduction regimen. Thus, no direct comparison of treatment efficacy of ART versus current first-line treatments for pain management can be made. By design, the AC group was not parallel in contact hours to the ART intervention. Although not methodologically ideal, the AC group showed essentially no reduction in overall pain, a finding we believe would have likely continued had additional control sessions been offered. However, random assignment was unblinded; hence, the potential existed for over-reporting of reductions in pain with the ART intervention. In addition, formal diagnoses of PTSD were not used; hence, results pertain to symptoms of PTSD and pain, and not diagnostic criteria. The study was conducted primarily among males and among those not in a psychological crisis, which limits broad generalizability. Finally, the present analysis is based on the acute effect of ART on pain reduction secondary to treatment of symptoms of PTSD. Long-term sustainability of results cannot be concluded from this analysis.

## Conclusions

This first controlled trial of ART for treatment of symptoms of combat-related PTSD substantiates a high prevalence of significant pain in US service members and veterans, including that of neuropathic origin, frequent head trauma, symptoms of concussion, or feeling dazed. Moreover, this analysis indicates that brief treatment with ART for symptoms of combat-related PTSD among service members/veterans also appears to have a marked generalizing effect to reductions in concomitant pain. Tailoring and future study of ART specific to pain management in service members and veterans appears warranted, as does mechanistic studies designed to identify how components of the ART protocol may reduce pain symptoms in conjunction with treatment of symptoms of combat-related PTSD.
